# The role of digital pharmaceutical governance in alleviating health inequality: a case study of the “Sanya Model” for centralized regional prescription review

**DOI:** 10.3389/fpubh.2026.1836281

**Published:** 2026-05-18

**Authors:** Xu Jia, Jun Xie, Fang Chai, HaoBo Jiang, Rui Shi

**Affiliations:** 1 Department of IT & Data Management of West China Hospital, Sichuan University, Chengdu, China; 2 Sanya People's Hospital, Sanya, China; 3 West China (Sanya) Hospital, Sichuan University, Sanya, China

**Keywords:** centralised e-prescription review, digital pharmaceutical governance, health equality, real-world evidence, tight-knit urban medical group

## Abstract

**Background:**

Irrational drug use poses a persistent challenge to healthcare quality and economic efficiency. In the context of China’s “Tight-knit Urban Medical Group” reform, this study analyzes a digitized pharmaceutical governance model designed to bridge the gap in pharmaceutical care between tertiary hospitals and primary healthcare facilities. We aimed to analyze the implementation and public health correlation of a Centralized E-prescription Review (CEPR) platform within a regional medical network.

**Methods:**

A retrospective, observational real-world study was conducted in Sanya, China. We implemented a CEPR platform utilizing a dual-layered cloud architecture and non-invasive data acquisition (XTL) to integrate regional drug metadata. The system incorporates an AI-driven engine fusing Large Language Models (LLMs) with Retrieval-Augmented Generation (RAG) to provide clinical decision support. A total of 441,327 prescriptions were analyzed over a 12-month longitudinal period. The Cochran-Armitage trend test was employed to evaluate temporal shifts in prescription safety, economic efficiency, and service homogeneity.

**Results:**

Following the implementation of the CEPR platform, the initial prescription pass rate at primary healthcare institutions rose significantly from 75.00 to 95.12%, and a 99.30% pharmacist intervention success rate was achieved across more than 16,000 intercepted high-risk orders. Regarding economic efficiency, the average cost per outpatient visit decreased by 4.58%, while inpatient drug costs dropped by 23.5%, accompanied by the implementation of a technology-based integrity oversight mechanism. Notably, the regional irrational prescription rate over 12 consecutive months of operation decreased markedly from 11.92 to 7.35% (*χ^2^* = 328.42, *p* < 0.001), demonstrating a robust temporal association between platform deployment and quality improvement.

**Conclusion:**

The CEPR model, characterized by the “Sanya Model,” serves as a digital lever for enhancing the accessibility and homogeneity of high-quality pharmaceutical services. By shifting medication safety from a site-specific resource to a standardized public service, this framework supports the “Three medical linkage” (San Yi Lian Dong, i.e., the integrated reform of medical services, health insurance, and pharmaceutical systems) collaborative reform. These findings provide a scalable governance paradigm for improving public health resilience in regions with unevenly distributed medical resources.

## 1 Introduction

Irrational drug use represents a formidable challenge to global healthcare systems. According to data from the World Health Organization (WHO), medication errors (MEs) contribute to approximately 2.6 million premature deaths annually, resulting in direct economic losses of about $42 billion—accounting for nearly 1% of the global total health expenditure [cf. (1)]. While e-prescription review has been proven effective in reducing transcription ambiguities and dosage inaccuracies [cf. (2, 3)], the evolution of healthcare toward an integrated health care delivery system—characterized by “online-offline coordination and multi-center linkage”—has exposed critical interoperability bottlenecks in traditional single-institution review models. These include difficulties in the homogenization of clinical decision support system (CDSS) rules, workflow congestion, and the persistence of data silos [cf. (4–6)].

International experience suggests that centralized e-prescription review (CEPR) is instrumental in enhancing the quality of medication use across entire healthcare networks. In the United States, pharmacy benefit management (PBM) systems have leveraged economies of scale to improve cost-containment efficiency; however, their lack of commercial transparency has sparked significant health equity debates [cf. (5, 7)]. In the United Kingdom, the NHS has utilized the enterprise master patient index (EMPI) to facilitate cross-institutional information sharing, yet remains lagging in rule adaptation and clinical pathway coordination for primary care [cf. (8, 9)]. Similarly, Saudi Arabia’s Wasfaty system has achieved national-level validation but still faces barriers in cross-departmental data traceability and the life-cycle management of prescriptions [cf. (10, 11)]. Consequently, a globally replicable technical paradigm that balances “regional standardization” with “institutional flexibility” remains elusive.

Since the promulgation of the Specifications for Prescription Review in Medical Institutions in 2018, China has mandated a “review-before-dispensing” protocol. Nevertheless, primary healthcare facilities have long suffered from a “structural poverty” in medication safety due to chronic shortages of clinical pharmacists and inadequate technological empowerment. Currently, the annual volume of prescriptions from China’s internet hospitals has exceeded 1.2 billion, covering 364 million online medical users. Traditional in-hospital review systems are increasingly inadequate for the trans-regional and virtualized characteristics inherent in internet medicine [cf. (12–14)].

As a national pilot city for public hospital reform, Sanya took the lead in launching a prescription oversight project based on algorithm-based governance by establishing a centralized e-prescription review (CEPR) platform. By integrating regional drug metadata and review rules, the platform innovatively utilizes non-invasive data acquisition tools (XTL) to achieve real-time data capture across heterogeneous systems. Furthermore, it marks the inaugural deep integration of pharmaceutical governance with integrity risk oversight, achieving a seamless closed-loop transition from “clinical risk control” to “regulatory compliance.” This exploration facilitates a strategic leap in public health management from an “institution-centered” model toward “municipal integration,” providing empirical evidence for the homogenization of health services.

## 2 Methods

### 2.1 Public health empowerment architecture of the platform

**Figure 1:**
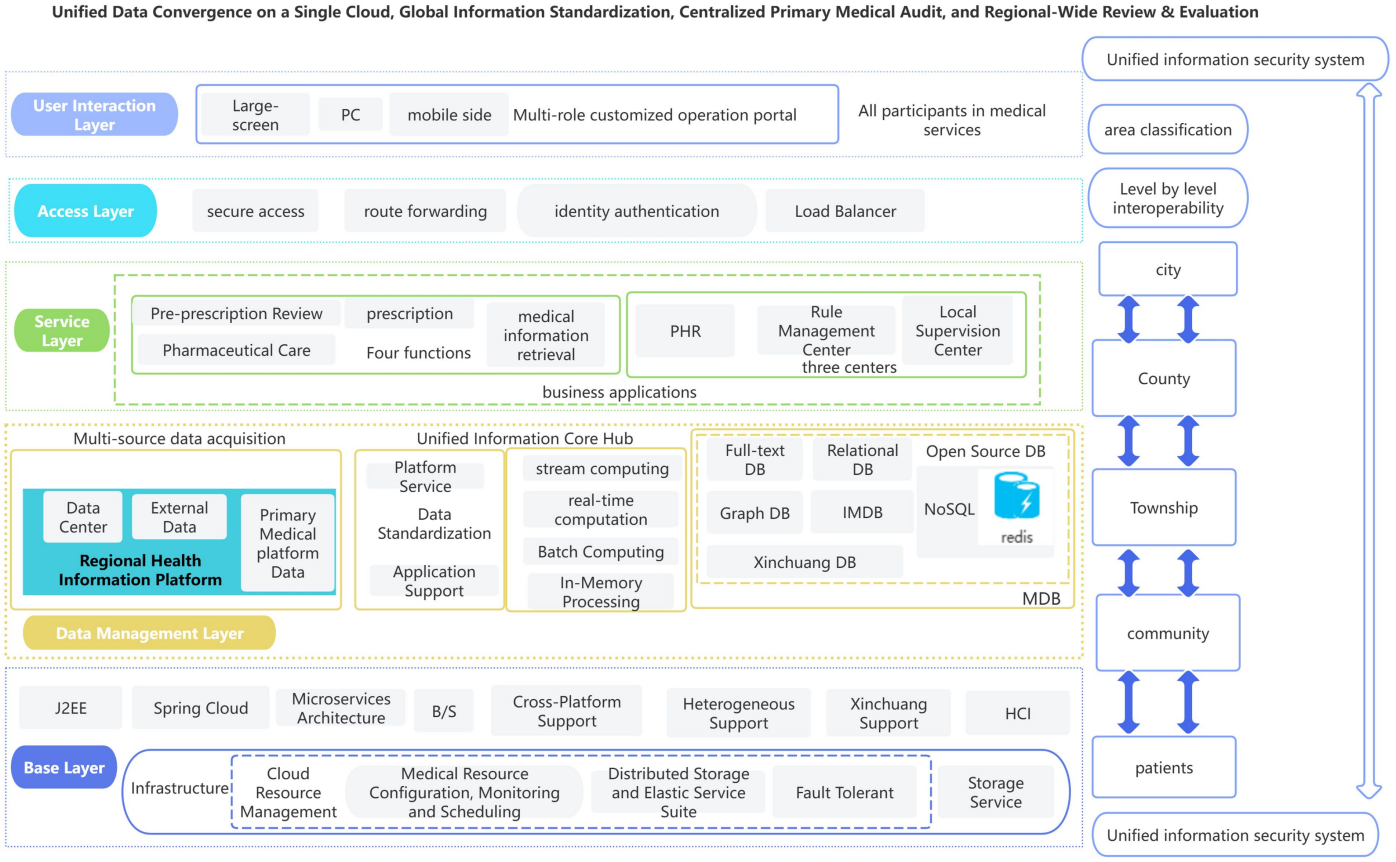
The overall architecture of the centralized e-Prescription Review.

The platform adopts a layered, modular microservices design based on Spring Cloud and Java2EE (Figure 1), ensuring data consistency and systemic response resilience in high-concurrency environments [cf. (5, 15, 16)]. The platform specifically constructs a public health governance framework characterized by “Three Major Centers and Four Core Functions” to achieve regional homogenization of management.

### 2.2 Construction of digital governance foundations via three supporting centers

#### 2.2.1 Master patient index (MPI) center

**Figure 2:**
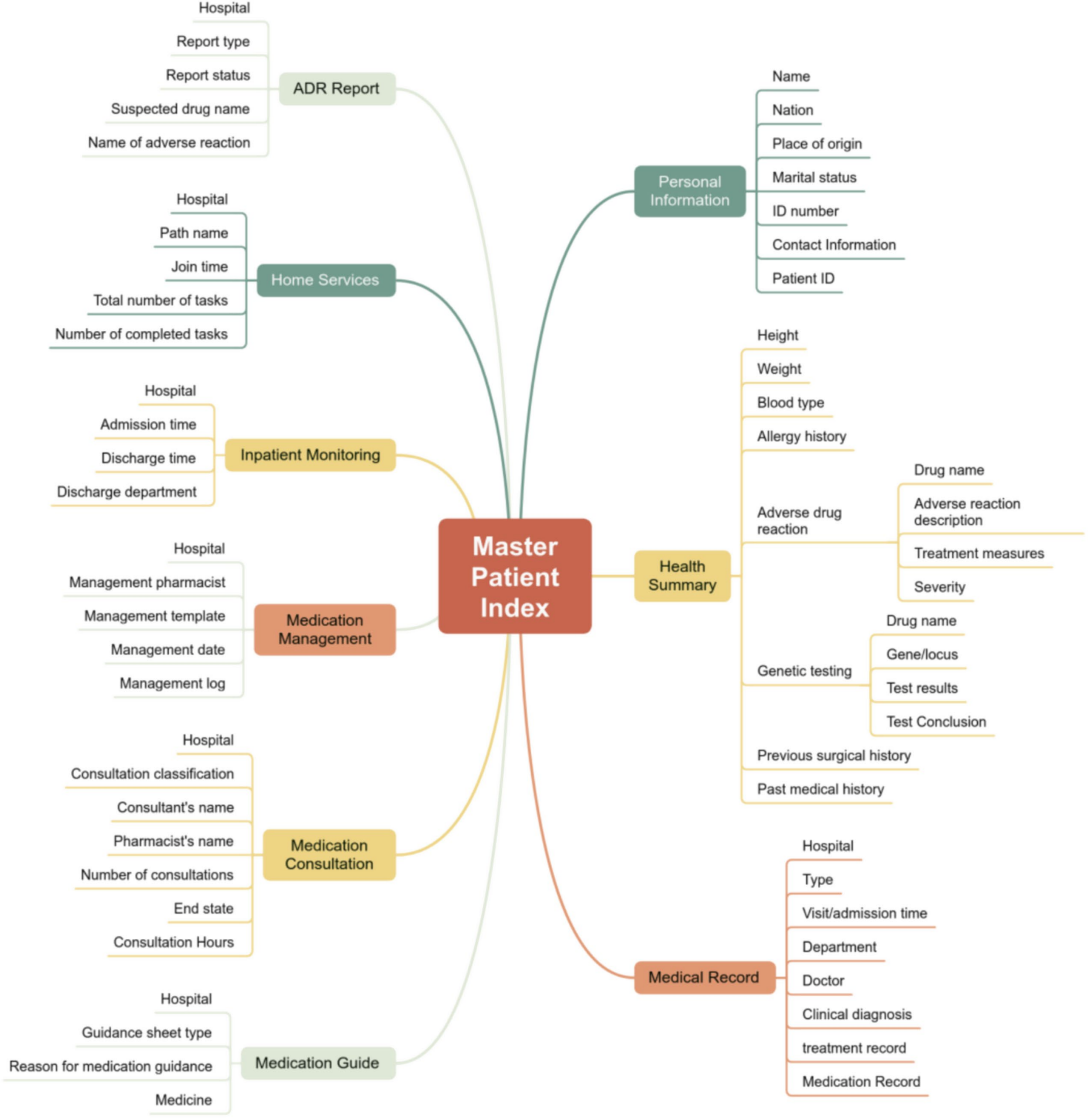
Master patient index.

Utilizing unique patient identifiers, the MPI center breaks down inter-institutional barriers by aggregating real-time clinical data—including medical history, allergies, and medication records—across different tiers of the medical group. This enables integrated cross-institutional screening for therapeutic duplication and drug-allergy contraindications, ensuring the continuity and depth of clinical interventions (Figure 2).

#### 2.2.2 Regional drug use monitoring center

**Figure 3:**
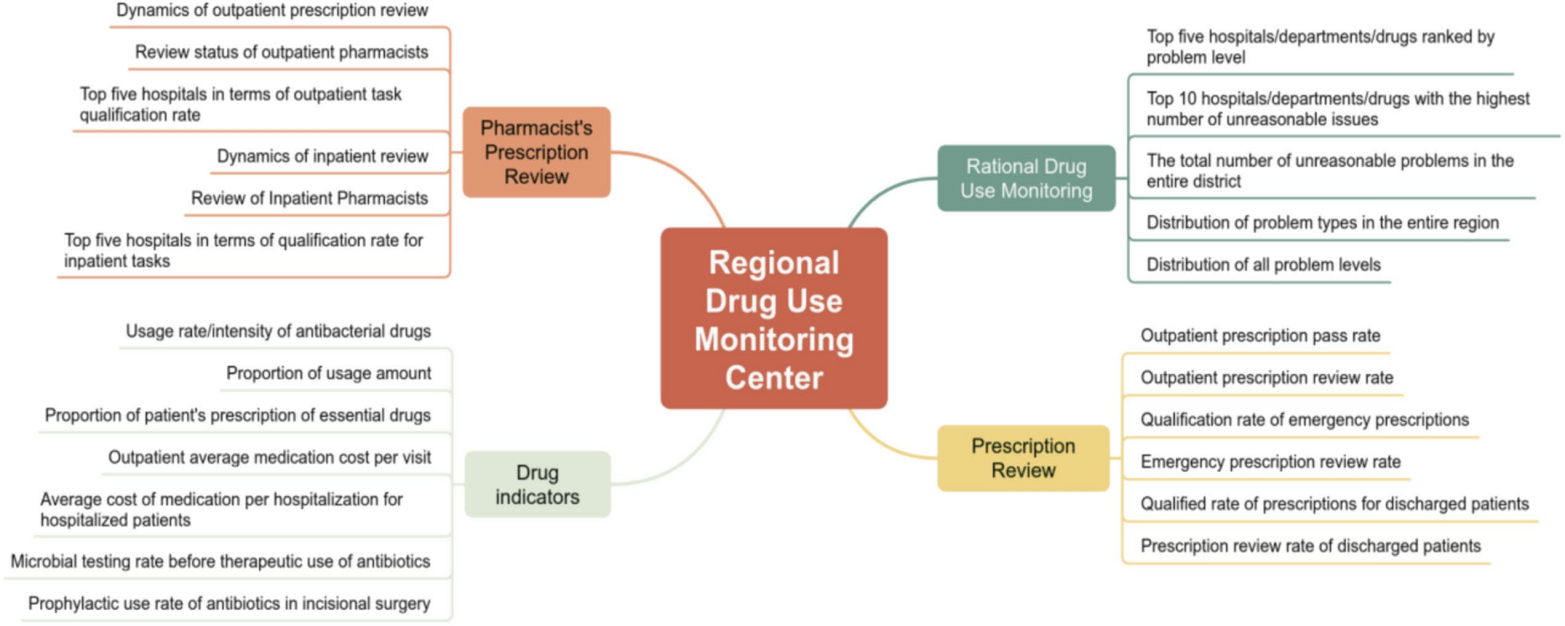
Regional drug use monitoring center.

This center features a real-time dynamic dashboard that displays regional medication quality indicators. It provides evidence-based insights for regional health policy-making, facilitating a strategic transition from passive supervision to proactive early warning (Figure 3).

#### 2.2.3 Dynamic rule management center

**Figure 4:**
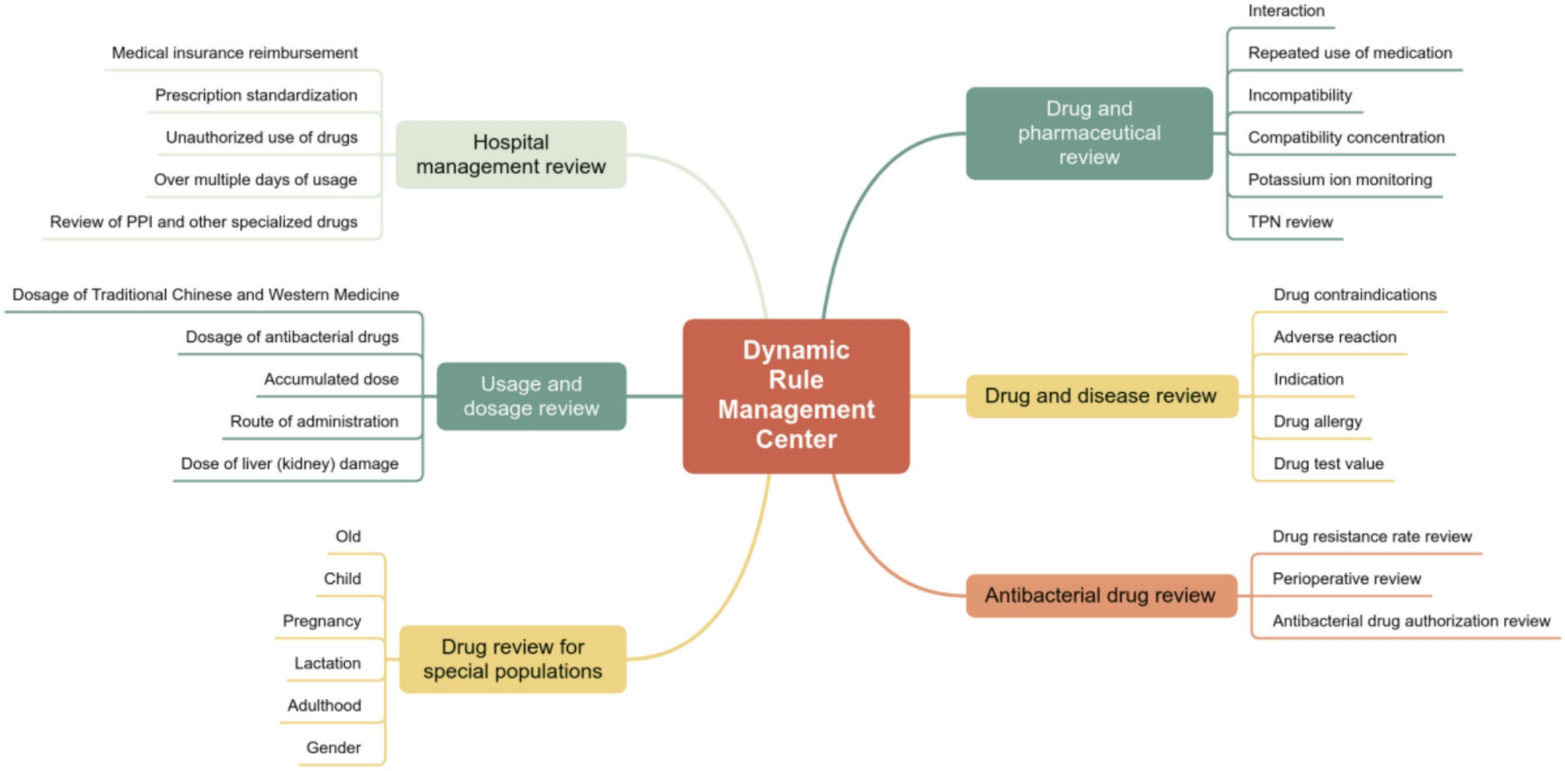
Dynamic rule management center.

The center integrates over 12 million evidence-based pharmaceutical rules covering 30 review dimensions. Through a strategy of “regional unified standards with institutional flexible configurations,” it ensures the homogenization of baseline safety standards across the medical group while granting necessary clinical flexibility to different specialties and tiers, embodying a governance logic of “standardization without rigidity” (Figure 4).

### 2.3 Four core functions empowering public health governance efficiency

#### 2.3.1 AI-driven intelligent rule engine and clinical decision support (CDSS)

**Figure 5:**
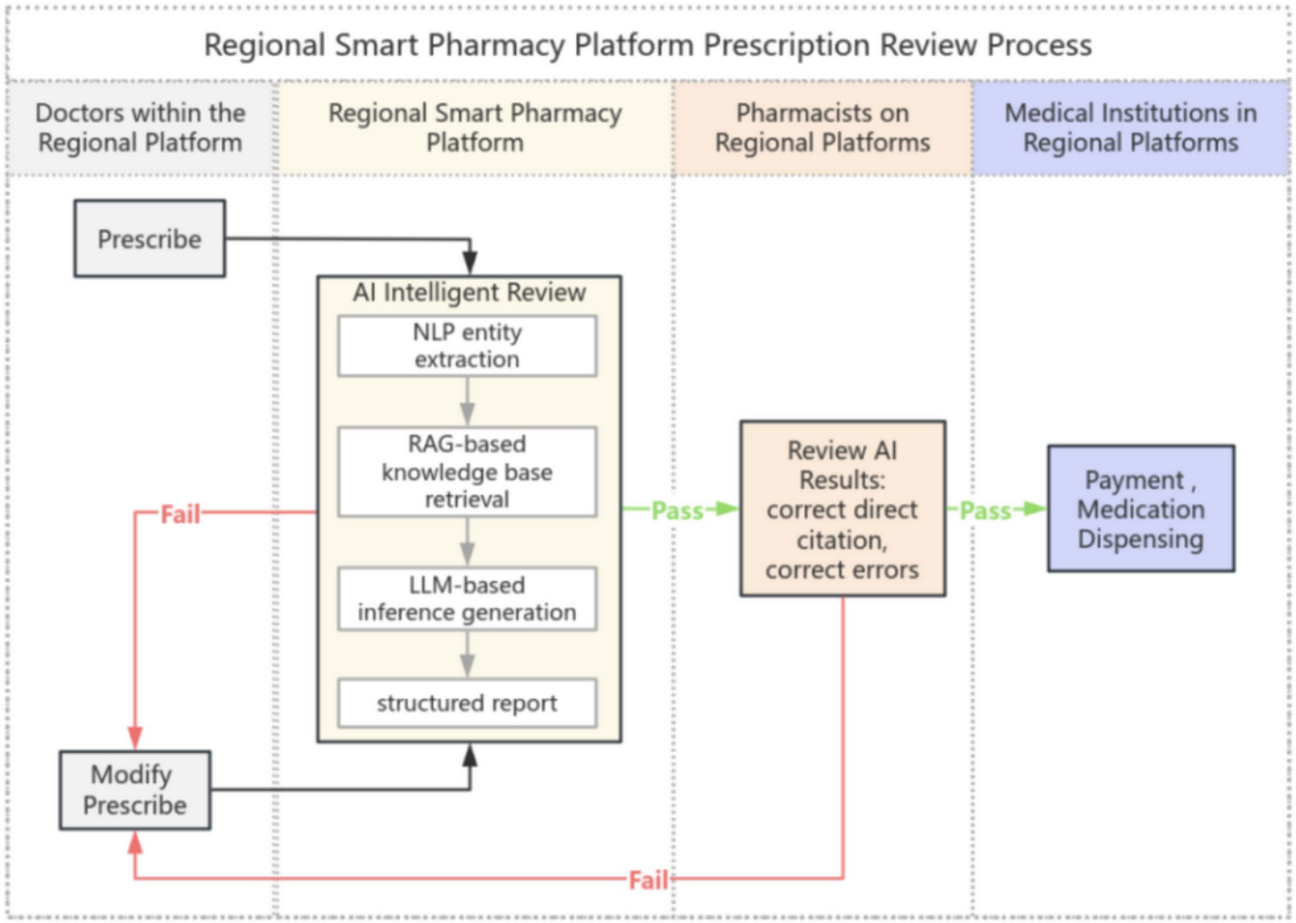
Prescription review process of regional review system.

The platform employs a self-developed AI engine integrating Large Language Models (LLMs, e.g., DeepSeek) with Natural Language Processing (NLP). To suppress “hallucinations,” the system utilizes Retrieval-Augmented Generation (RAG) to anchor LLM reasoning within structured clinical guidelines, optimized through domain-specific prompt engineering and knowledge base embeddings (e.g., Chinese National Formulary 2020 and Administrative Specifications for Long-term Prescriptions 2021). This framework demonstrated >90% agreement with independent pharmacist reviews in a manual validation (*N* = 200). Clinically, the engine performs millisecond-level automated screening (Figure 5), providing real-time support that bridges the expertise gap among primary care physicians. This high-throughput model enables regional pharmacists to oversee vast populations efficiently, ensuring that remote village clinics come close to the same safety standards and service homogeneity as tertiary hospitals in Sanya [cf. (4, 5)].

#### 2.3.2 Real-time multi-tier intervention and closed-loop interception

The system implements differentiated interventions based on risk levels (Red, Yellow, and Green lights). High-risk prescriptions are subject to mandatory interception, requiring physicians to either rectify the order or provide robust clinical evidence. This proactive risk-blocking mechanism eliminates potential medication errors (MEs) at the source, effectively reducing irrational medication-related public health expenditure and the loss of Disability-Adjusted Life Years (DALYs).

#### 2.3.3 Centralized remote pharmacist review and resource redistribution

For complex risks identified by AI, prescriptions are automatically routed to a cloud-based review center. This bypasses geographical constraints, allowing expert pharmacists to provide remote guidance to primary care facilities. This process facilitates the digital “downward shift” of premium resources and reshapes the supply-side structure of regional pharmaceutical services, provides a potential structural solution for the global healthcare workforce shortage (Figure 5).

#### 2.3.4 Integrity oversight and regulatory compliance linkage

The platform innovatively synchronizes operational data to a disciplinary supervision module in real-time using XTL tools. By generating automated alerts for abnormal medication frequencies, it achieves “technological anti-corruption.” This integration significantly enhances the transparency of the healthcare system and safeguards the integrity of the health insurance fund, demonstrating the value of digital tools in broader social governance.

### 2.4 Study setting and participants

This retrospective study focused on 239,557 primary care patients recorded within a centralized regional prescription review system across 33 healthcare facilities (including 8 branch hospitals and 25 village clinics). Descriptive statistics were employed to characterize the study sample.

### 2.5 Definitions of key indicators

#### 2.5.1 Irrational prescription

Following the “Specifications for Prescription Review in Medical Institutions” issued by the National Health Commission of China, an “Irrational Prescription” is defined as any medication order that fails to comply with the criteria of legitimacy, standardization, or clinical appropriateness. The evaluation framework comprises 20 sub-categories (detailed in Table 1), encompassing issues such as off-label usage, inappropriate dosing, therapeutic duplication, and clinically significant drug–drug interactions. The underlying rule base is synthesized from manufacturer drug inserts, the National Formulary, and evidence-based clinical practice guidelines.

**Table 1:** Classification of prescription review criteria.

Category	Key review points
Legality and standardization	Missing mandatory fields in the prescription header, body, or footer; prescribing beyond authorized scope; substandard notation of dosage and administration instructions.
Clinical appropriateness	Off-label use; inappropriate dosage range (exceeding limits for a single dose, daily dose, or total course duration); inappropriate route of administration.
Safety risks	Therapeutic duplication (identical ingredients or similar pharmacological mechanisms); drug–drug interactions (antagonistic effects or cumulative toxicity); contraindications (age, gender, or disease-specific).
Special populations	Pediatric dosing not calculated based on body weight; Potentially Inappropriate Medication (PIM) for the older adult; failure to adjust dosage for patients with impaired hepatic or renal function.
Clinical monitoring	Prescribing medications in the absence of necessary biochemical monitoring (e.g., potassium levels, coagulation profiles, etc.).

#### 2.5.2 Initial prescription pass rate

Initial Prescription Pass Rate is defined as the proportion of prescriptions that successfully pass the automated system screening or trigger alerts but are subsequently approved by the pharmacist before the manual review stage.

#### 2.5.3 Successful pharmacist intervention

Successful Pharmacist Intervention refers to the process where a prescription is identified as non-compliant by the system—excluding those voluntarily withdrawn/modified by the physician or directly intercepted by the system—and a pharmacist provides clinical recommendations which the prescribing physician subsequently accepts and implements. The process is only deemed successful once the modified prescription is re-audited and approved by the pharmacist.

### 2.6 Statistical analysis

Data were analyzed using the Cochran-Armitage trend test to evaluate the dynamic evolution of irrational drug use rates over 12 months (*N =* 441,327, A total population sampling approach was used to analyze all eligible prescriptions within the study period). The precision of estimates for key performance indicators (KPIs) was measured using 95% confidence intervals (CIs). All statistical inferences were based on two-sided tests, with a *p* < 0.05 considered statistically significant.

## 3 Results

### 3.1 Characteristics of the study sample

The demographic and clinical profiles of the population covered by the platform are summarized in Table 2. The sex distribution was nearly balanced, with a male-to-female ratio of approximately 1:1.21. Regarding age stratification, young and middle-aged adults (18–40 years) constituted the largest cohort (35.93%), while the older adult population (≥60 years) accounted for 31.62%.

**Table 2:** Sociodemographic characteristics and morbidity profiles of the study population.

Category	Indicator	Count	Percentage
Gender	Male	108,496	45.29%
	Female	131,061	54.71%
Age group	18–40	77,747	32.45%
	40–60	86,069	35.93%
	≥60	75,741	31.62%
Major diseases	Hypertension	29,576	12.35%
	Diabetes	10,291	4.30%
	Pneumonia	8,463	3.53%
	Skin disease	6,338	2.65%
	Arthritis	3,912	1.63%
	Neoplastic disease	459	0.19%
	Stroke	312	0.13%

Analysis of morbidity patterns identified hypertension (*n* = 29,576, 12.35%), diabetes (*n* = 10,291, 4.30%) and pneumonia (*n* = 8,463, 3.53%) as the predominant chronic conditions. This distribution aligns with the established public health priorities and mortality risk rankings for Sanya residents, consistent with the Sanya Statistical Yearbook 2024 and the study by Zhu Mingsheng et al. [cf. (17)]. The high prevalence of these chronic diseases, particularly among the older adult demographic, underscores the necessity for synchronized and homogenized pharmaceutical care at primary healthcare facilities through the CEPR platform.

### 3.2 Narrowing healthcare disparities and enhancing medication safety

**Figure 6:**
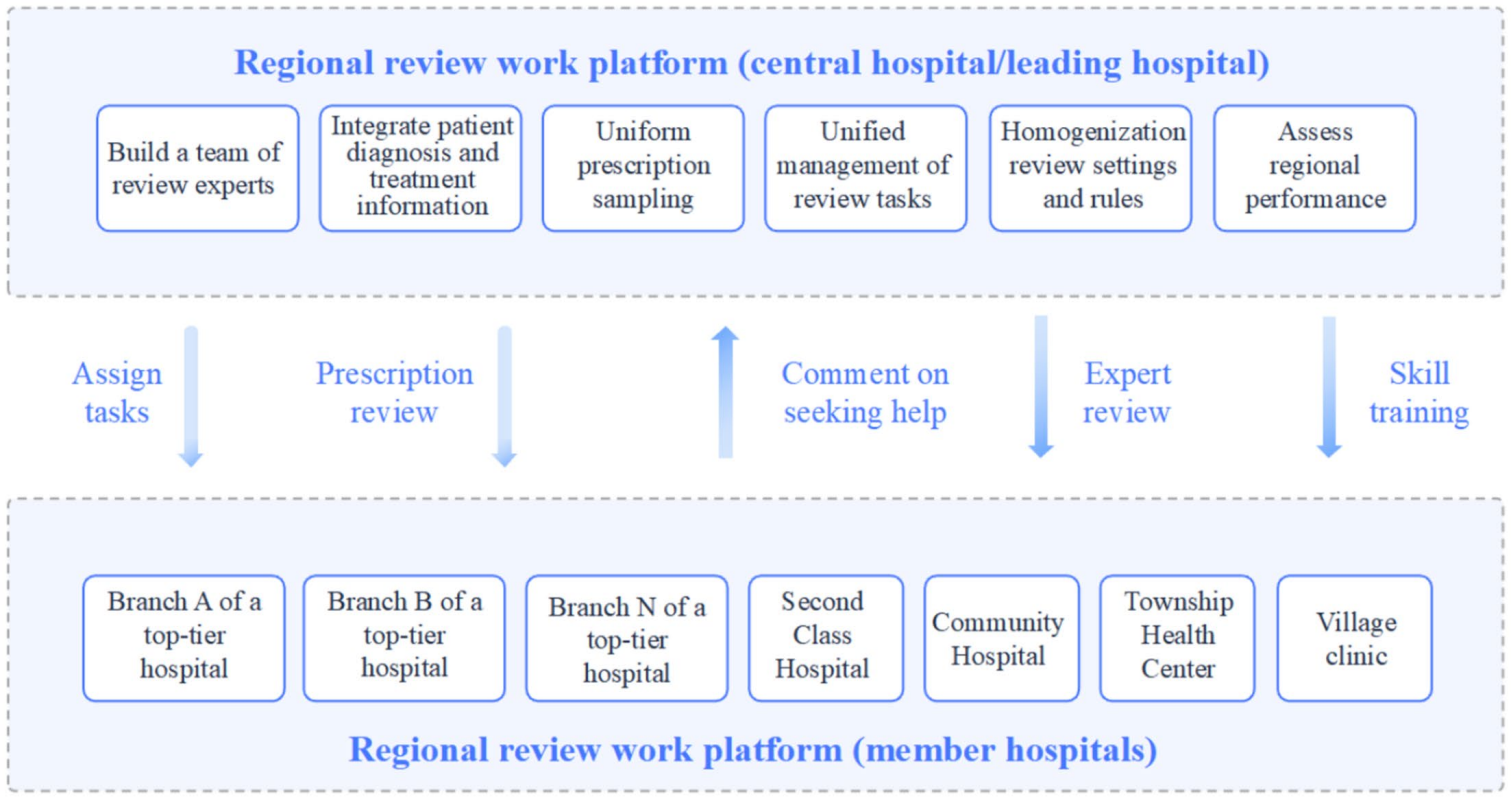
Thea Dual-tier collaborative mechanism for prescription review and clinical auditing.

Following the platform’s deployment, a dual-tier collaborative mechanism for prescription review and clinical auditing was established—directly linking the central platform with primary healthcare providers—encompassing one central tertiary hospital located in the urban district, 8 branch hospitals, and 25 village clinics (Figure 6). This framework enables primary healthcare facilities to receive technical support and professional training from superior hospitals. Additionally, the system delivers pharmaceutical education and follow-up reminders to patients, significantly improving the awareness of rational drug use and overall patient satisfaction.

**Figure 7:**
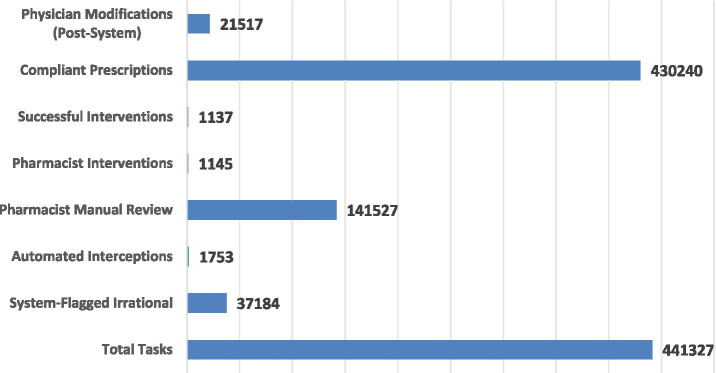
CEPR implementation from November 2024 to October 2025.

From November 2024 to October 2025, the system audited a total of 441,327 prescriptions. Among these, 37,184 tasks were identified as irrational by the automated system, leading to 21,517 modifications by physicians following the review. Pharmacists ultimately intercepted nearly 16,000 high-risk irrational prescriptions, achieving a successful intervention rate of 99.30%. Notably, the initial prescription pass rate at primary healthcare institutions significantly increased from 75.00 to 95.12% (Figure 7), demonstrating a substantial homogenization of medication safety standards across the region.

**Figure 8:**
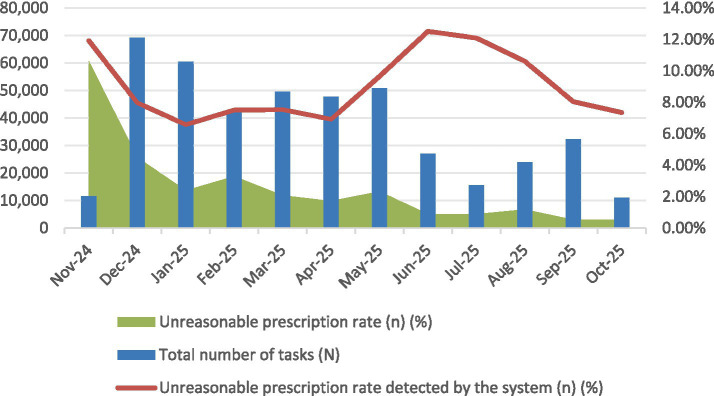
Monthly trend chart of prescription quality.

Over the 12 months of stable operation since its deployment, the platform has processed a total of 441,327 prescription tasks (Table 3; Figure 8). Statistical analysis revealed that the regional rate of irrational prescriptions decreased significantly from 11.92% (95% *CI:* 11.75–12.09%) to 7.35% (95% *CI:* 7.21–7.49%). The Cochran-Armitage trend test yielded *χ^2^* = 328.42, *p* < 0.001, confirming that the observed quality improvement is statistically highly significant and demonstrating a robust “time-effect” correlation between platform implementation and the enhancement of medication quality.

**Table 3:** Statistical significance of key indicators (*N =* 441,327).

Phase	Total tasks (*N*)	Irrational rate (*P*) [95% *CI*]	Pharmacist intervention (R) [95% *CI*]
Baseline (11.2024)	11,634	11.92% [11.3–12.5]	0.00% [0.0–0.0]
Running phase (12.2024–09.2025)	418,716	8.36% [8.2–8.5]	3.25% [3.1–3.4]
End phase (10.2025)	10,977	7.35% [6.9–7.8]	0.87% [0.3–1.4]
Trend test	—	Ptrend < 0.001	—

### 3.3 Reducing patient burden and improving health economic efficiency

**Figure 9:**
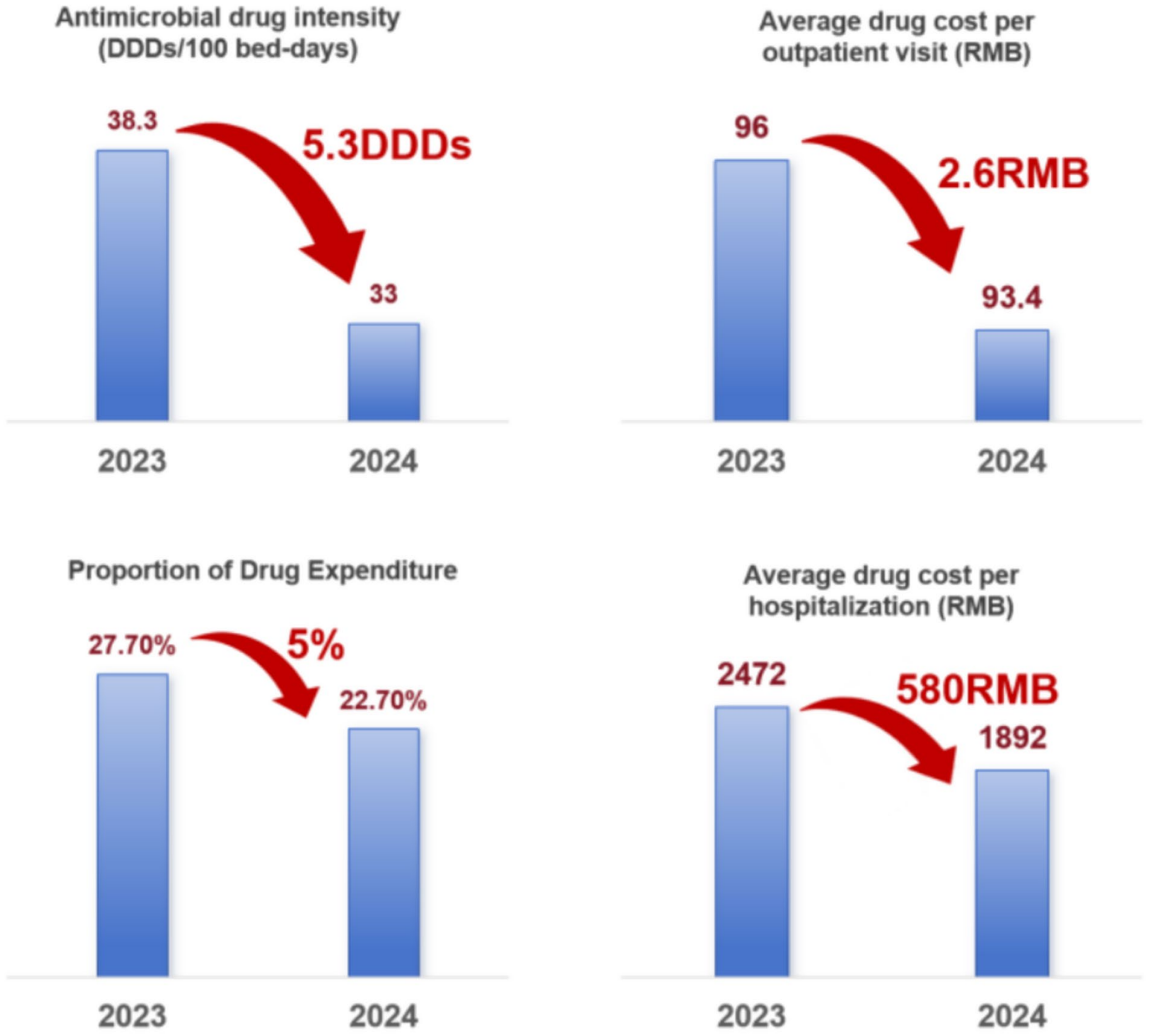
Reduction of some medical costs.

This study employed a “pre- and post-intervention direct cost comparison method” to evaluate the economic impact of the CEPR platform. Core economic indicators, such as the average drug cost per outpatient visit and per inpatient stay, were extracted directly from the internal administrative audit reports of the Sanya Health Commission. Comparing the data from 2023 and 2024, the results demonstrate that centralized e-prescription review (CEPR) successfully optimized the pharmaceutical expenditure structure, achieving a concurrent reduction in both outpatient and inpatient costs (with an average saving of 2.6 RMB per outpatient visit and 580 RMB per inpatient admission). Specifically, the average cost per outpatient visit decreased by 4.58% year-on-year, while inpatient drug costs dropped by 23.5%, and the drug-to-total-cost ratio (drug markup) declined by 5 percentage points. By intercepting irrational prescriptions, the system effectively mitigated the financial burden on patients and alleviated expenditure pressure on the health insurance fund. Furthermore, the platform successfully regulated the Defined Daily Dose (DDD) per 100 bed-days for antimicrobials, directly reducing the collective public health risk associated with community-acquired antibiotic resistance (Figure 9).

### 3.4 Integration of medical governance and ethical risk control

**Figure 10:**
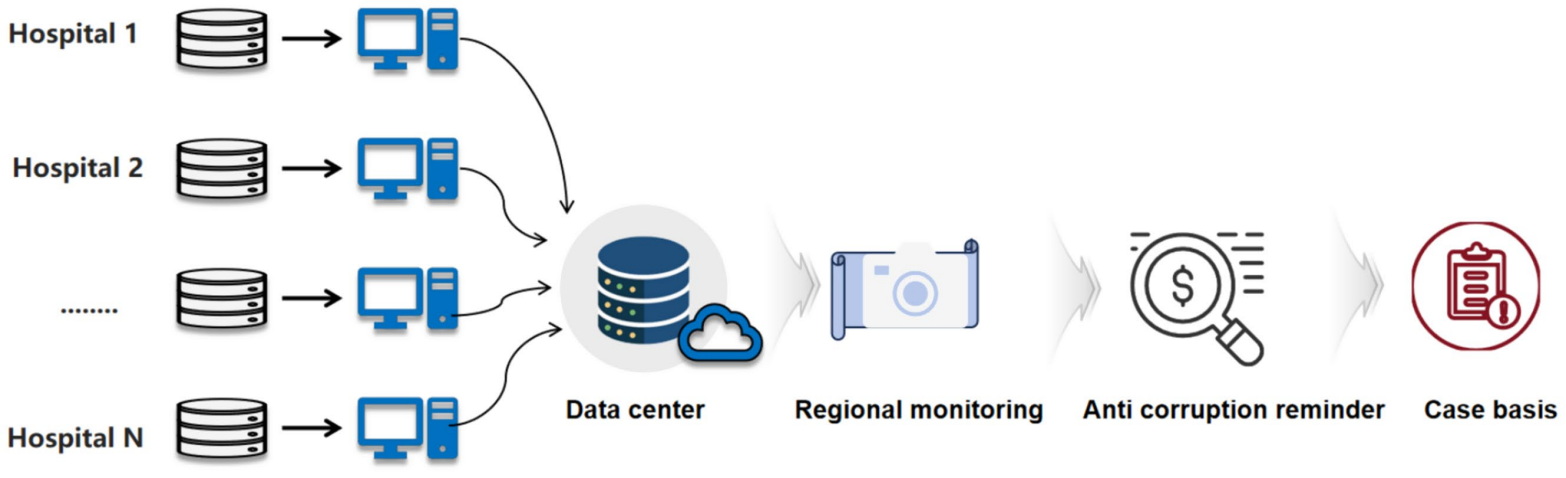
Real-time monitoring of prescription access through discipline inspection and supervision.

The platform established a collaborative integrity risk oversight mechanism linked with disciplinary supervision (Figure 10). This system generates automated, real-time alerts for the abnormal prescription of key monitored drugs. Since 2024, 50 compliance leads have been automatically routed and resolved (with a completion rate of 96.1%). This mechanism has effectively deterred the “kickback-based” pharmaceutical sales chains, highlighting the strategic value of digital tools in the broader context of public health social governance.

## 4 Discussion

This study constructed and evaluated a Centralized E-prescription Review (CEPR) platform within the context of a tight-knit urban medical group. The platform fundamentally represents a digital redistribution mechanism for pharmaceutical resources. By utilizing algorithms to transmit high-quality medication literacy from tertiary hospitals to the periphery of the healthcare system, it transforms “medication safety” from a site-specific, scarce resource into a standardized public good. This shift effectively safeguards the health rights of vulnerable populations.

### 4.1 Optimization of resource allocation and enhancement of service homogeneity

The platform processes an average of 36,777.25 prescriptions per month. This high-throughput capability demonstrates that a consolidated team of regional pharmacists can effectively oversee medication safety for an expansive population (*N* = 239,557). Compared to traditional decentralized review models, this centralized approach represents a more sustainable allocation of human resources, mitigating the heavy reliance of primary healthcare institutions on dedicated, high-level clinical pharmacists. Concurrently, by leveraging a unified AI rule library and standardized regional pharmacist review protocols, the system ensures that patients receive an equivalent standard of medication safety regardless of their point of care. This effectively narrows the service quality divide between urban and rural institutions and enhances the equitable accessibility of high-quality pharmaceutical care.

### 4.2 Endogenous governance drivers via “institution-technology-talent” synergy

The core contribution of this study lies in the construction of a tripartite synergistic system that ensures digital interventions transition from experimental environments to routine public health practice. At the institutional level, linking review results rigidly with performance evaluations and disciplinary supervision resolves the “authority-responsibility dilemma” in traditional pharmaceutical services, where advisory power often outweighed decision-making power. At the technical level, the system relies on a repository of 900,000 rules and a 0.4-s response speed to provide reliable Clinical Decision Support (CDSS) in high-concurrency scenarios. At the talent level, standardized empowerment enhances the professional capacity of pharmacists across the entire regional network.

### 4.3 A health economic governance paradigm under the “three medical linkage”

The platform serves as a critical lever for the “Three medical linkage” reform involving medical services, health insurance, and pharmaceuticals. On the medical and insurance side, real-time interception of irrational prescriptions directly reduces drug expenditure and provides precise real-world data (RWD) support for payment reforms such as DRG/DIP. On the pharmaceutical side, big data analytics based on medication profiling provides evidence-based insights for Group Purchasing Organizations (GPO) and drug selection. This transition achieves a profound integration of micro-level clinical interventions with macro-level health economic governance.

### 4.4 Balancing homogenization and flexibility to bridge the “quality gap”

To address the “structural poverty” of uneven regional medical capabilities, this study implemented a stratified and flexible intelligent supervision strategy. A “one-size-fits-all” mandatory interception is applied to extreme-risk prescriptions to ensure a safety baseline; for low-to-moderate risks, differentiated authorization preserves the clinical flexibility of primary care physicians. This governance logic of “standardization without rigidity” effectively bridges the quality gap in pharmaceutical services between urban and rural areas, serving as a technical guarantee for enhancing the accessibility and homogeneity of high-quality pharmaceutical services.

### 4.5 Organizational reshaping and ethical synergy under digital transformation

The CEPR platform has driven a deep reshaping of healthcare organizations. In terms of professional transition, AI filters over 90% of routine logic errors, releasing pharmacists from low-value “mechanical verification,” alleviating alert fatigue, and facilitating their evolution into “Advanced Medication Therapy Management Experts.” Regarding organizational ethics, “algorithm-based governance” creates a transparent environment based on data evidence rather than administrative authority, enhancing internal trust. Simultaneously, by maintaining the pharmacist’s final decision-making power (human-in-the-loop), the system ensures a balance between technical rigidity and humanistic care.

### 4.6 Paradigmatic evolution from clinical tool to social governance instrument

Compared to existing regional prescription review practices globally, this study achieves paradigmatic innovation in two dimensions, elevating the system beyond a mere clinical tool into a public health instrument with broad social governance implications.

One is Architectural and Integration Innovation: The adoption of a dual-layered “Front-end + Center” architecture and non-invasive XTL tools overcomes international technical barriers, such as the difficulty of integrating heterogeneous systems in primary care and performance bottlenecks during high-concurrency.

The second is Governance Dimension Innovation: This is one of the world’s earliest cases to deeply link pharmaceutical risk control with integrity monitoring, utilizing technical means to curb improper interest associations within the pharmaceutical supply chain.

### 4.7 Limitations and future outlook

First, the multicenter generalizability of the model remains to be validated. The findings are anchored in the specific regional healthcare environment of Sanya’s integrated medical community, characterized by unique policy support and informatics infrastructure. Consequently, caution is warranted when extrapolating the “Sanya Model” to regions with divergent healthcare resource levels or administrative frameworks. Moreover, due to the lack of an external parallel control group (as data were derived from internal system logs and administrative records), a definitive causal relationship between platform implementation and clinical outcomes has not been established, despite a strong temporal association. Future prospective, large-scale, multicenter studies are essential to validate the model’s efficacy across regions with diverse economic and infrastructural profiles.

Second, as a retrospective observational study, selection bias is inherently unavoidable. The dataset primarily originated from pilot institutions integrated into the CEPR platform, which may not fully represent the prescribing patterns of non-pilot facilities with differing resource allocations or IT infrastructures. Additionally, the adaptation period for pharmacists during initial deployment and variations in data recording completeness may have introduced minor biases into early-stage assessments. Furthermore, it remains challenging to isolate the independent effects of the intervention from external confounders, particularly national Group Purchasing Organizations (GPO) and health insurance payment reforms (e.g., DIP/DRG). Given the absence of a randomized controlled design, the observed cost reductions should be interpreted as the result of multi-factorial synergy rather than being solely attributed to platform interventions.

Third, as this study was conducted against the macro backdrop of national GPO and payment reforms, we could not employ rigorous econometric models to decouple the systemic influence of these policies on drug unit prices and total expenditures. Therefore, the observed decline in average prescription costs cannot be entirely credited to the CEPR platform. Future research should utilize controlled designs or regression discontinuity methods to quantify the respective contributions of platform-specific interventions versus policy-driven cost reductions.

Fourth, the applicability of the system in complex clinical scenarios is yet to be fully determined. Current evaluations primarily cover outpatient and internet hospital prescriptions. The system’s response latency, rule complexity, and real-time integration with physiological parameters have not been rigorously assessed in high-dynamic, urgent, or multidisciplinary environments, such as Emergency Departments and Intensive Care Units (ICUs).

Finally, there is a lack of in-depth quantitative assessment regarding the governance dimensions. While the linkage between pharmaceutical risk control and disciplinary supervision has been established, the long-term impact of this “technological anti-corruption” mechanism on physicians’ prescribing behaviors and their underlying psychological motivations remains unexplored through systematic qualitative or longitudinal analysis.

## 5 Conclusion

Addressing the global challenges of irrational drug use and the inequitable distribution of medical resources, this study innovatively constructed a Centralized E-prescription Review (CEPR) platform—characterized by its “Three Major Centers and Four Core Functions”—within the context of China’s tight-knit urban medical group reform. By leveraging a dual-layered cloud architecture (“Front-end Review + Regional Center”) and non-invasive data acquisition technology, the platform achieved homogenized pharmaceutical oversight across a four-tier healthcare network encompassing municipal, county, township, and village levels.

Empirical evidence indicates that following the platform’s implementation, the initial prescription pass rate at primary healthcare facilities surged from 75.00 to 95.12%. The pharmaceutical expenditure structure for both outpatient and inpatient services was significantly optimized, with a successful pharmacist intervention rate of 99.30% achieved across 441,327 real-world prescriptions. This model not only effectively bridged the “quality gap” in urban–rural health services through the synergy of AI and remote pharmacists, but also mitigated systemic governance bottlenecks in the “Three medical linkage” by deeply integrating pharmaceutical risk control with disciplinary supervision. Consequently, the “Sanya Model” provides a highly valuable digital governance paradigm for enhancing public health resilience, promoting the accessibility and homogeneity of high-quality pharmaceutical services, and ensuring the security of the health insurance fund.

## Data Availability

The raw data supporting the conclusions of this article will be made available by the authors, without undue reservation.
